# Regular assessment of serum vascular endothelial growth factor levels to monitor POEMS syndrome

**DOI:** 10.1007/s10072-023-07064-5

**Published:** 2023-09-13

**Authors:** Francesco Gentile, Fabrizia Terenghi, Pietro Emiliano Doneddu, Alberto De Lorenzo, Claudia Giannotta, Andrea Giordano, Rita Mazza, Andrea Nozza, Eduardo Nobile-Orazio

**Affiliations:** 1 Neuromuscular and Neuroimmunology Unit, Department of Medical Biotechnology and Translational Medicine, IRCCS Humanitas Research Hospital, Milan University, Via Manzoni 56, 20089 Rozzano, Italy; 2 Neurology Residency Program, University of Milan, 20122 Milan, Italy; 3 Department of Biomedical Sciences, Humanitas University, Via Rita Levi Montalcini 4, 20090 Pieve Emanuele, Rozzano, Italy; 4 Department of Medical Oncology and Hematology, Humanitas Cancer Centre, Humanitas Clinical and Research Hospital IRCCS, Rozzano, Italy; 5 Department of Medical Biotechnology and Translational Medicine, University of Milan, Milan, Italy

**Keywords:** POEMS syndrome, VEGF, Biomarker, Neuropathy, Monitoring, Relapse

## Abstract

**Background:**

To investigate the utility of regular serum VEGF (sVEGF) levels assessment in the monitoring of POEMS syndrome.

**Methods:**

We retrospectively reviewed data of 30 patients with POEMS syndrome whose sVEGF was tested regularly every 6 months. sVEGF levels after treatment were measured and correlated with disability (Overall Neuropathy Limitations Scale, ONLS), clinical impairment (measured with the modified Clinical Response Evaluation Scale, mCRES), and relapse-free survival. The ability of sVEGF to predict disease flares during remission and refractory disease was also analysed.

**Results:**

Patients with normalised serum VEGF levels (< 1000 pg/ml) at 6 months showed prolonged relapse-free survival (at 3-year 94% for complete VEGF response, 57% partial, 0% none, *p* < 0.001) and greater later clinical improvement (median ΔmCRES complete VEGF response -5 vs partial -4, *p* = 0.019, and vs no VEGF response -2, *p* = 0.006). After remission, the sensitivity of 6-month sVEGF monitoring in predicting clinical relapse was 58% with a specificity of 100%. In patients refractory to treatment, the sensitivity in predicting further clinical worsening was 15%. In addition, in 25% of the patients in remission and 16% of those refractory to therapy, sVEGF levels only increased at the time of relapse.

**Conclusions:**

Regular sVEGF assessment is a valid biomarker in the prediction of disease reactivation in POEMS syndrome and was particularly useful during the phase of remission.

## Introduction

POEMS (polyneuropathy, organomegaly, endocrinopathy, monoclonal gammopathy, and skin changes) syndrome is a multisystemic disorder defined by the presence of peripheral neuropathy and monoclonal plasma cell disorder [1, 2]. The diagnosis requires fulfilment of specific clinical, imaging, and biochemical criteria, including elevated levels of vascular endothelial growth factor (VEGF) detected in more than 85% of cases [1, 3, 4].

VEGF is a cytokine which stimulates angiogenesis and vascular permeability [5] and is deemed to be pathogenically associated to some of the classical features observed in POEMS syndrome, including neuropathy, extravascular overload, haemangiomas, and papilledema [6]. It is mainly secreted by bone marrow plasma cells, with nearby polyclonal rather than monoclonal cells being responsible for its production [7, 8]. Other cells including platelets may also contribute to the total circulating levels [9]. VEGF measured in either serum or plasma is essential in the diagnostic work-up of POEMS syndrome, since it demonstrates high sensitivity and specificity for the disease [10, 11] and its early testing reduces excessive costs and time delay in the diagnosis [12].

Several case series suggest that VEGF correlates with disease activity [13, 14], but the reported discrepancies between measured levels and relapses [15, 16], and the raise of VEGF in other illnesses [10, 11, 17–19], raise some doubt on the utility of monitoring VEGF during the follow-up of POEMS patients. Some recent studies suggest however that monitoring VEGF levels may help in predicting worsening in patients with POEMS syndrome [20, 21].

Taking advantage of a large collection of regular serum VEGF (sVEGF) measurements in a cohort of 30 patients with POEMS syndrome, we investigated the utility of regular sVEGF assessment in monitoring POEMS patients. We also evaluated whether sVEGF levels at 6 months and 1 year after therapy predicts long-term clinical improvement and remission.

## Materials and methods

### Study population and design

We retrospectively reviewed the disease course of patients with POEMS syndrome who were monitored with a regular sVEGF assessment (every 6 months), followed at our institution (Humanitas Research Institute) between October 2004 and June 2022. Clinical and demographic data were retrieved from hospital charts and outpatient visits. A subset of cases had been already included in a previously published prospective open-label trial performed in our institution [22]. A multisystemic diagnostic assessment was employed in all patients, including clinical/neurological evaluation, laboratory markers, and imaging studies, and the diagnosis of POEMS syndrome was reached according to valid criteria [1]. After treatment, patients underwent regular neurologic and haematologic assessments and regularly monitored for sVEGF levels [10], tested before therapy initiation and every 6 months after therapy. sVEGF levels were determined by ELISA using a commercially available system (Research & Diagnostic Systems, Minneapolis, MN). We included in the study patients with at least 1 year of follow-up in case of response to treatment (*n* = 27) and also patients who relapsed within 12 months (*n* = 3) to avoid potential missing of informative data in these cases. Approval by Ethic Committee is not required as this is a retrospective study on data and test routinely collected during regular clinical activity. All the patients admitted to the hospital had signed a consensus for the use of their clinical and laboratory results for research purpose.

Ability of 6-month monitoring of sVEGF levels to predict clinical relapse during remission or further clinical deterioration during refractory disease was analysed. The ability of sVEGF to predict clinical worsening was separately calculated for therapies that had induced remission and those without a clinical response (refractory disease). sVEGF levels at 6 months and 1 year after treatment were measured and correlations with disability, complete remission, and relapse-free survival were calculated. Original data can be shared with other authors upon reasonable request.

### Definition of remission, refractory disease, and clinically meaningful change

Remission was defined as a period of clinical stability or improvement (measured with ONLS and mCRES, see below) following treatment that had induced a normal immune fixation. Refractory disease was defined as an absence of clinical improvement after therapy that required a change of treatment and not related to intolerance to the therapy. Clinical meaningful change was considered in the presence of at least one of these conditions: (1) *neurological*: change of ≥ 1 point on the Overall Neuropathy Limitation Scale (ONLS, score 0 (normal) to 12 (maximal impairment)) [23, 24] and (2) *clinical*: change of ≥ 2 points of a modified version of the Clinical Response Evaluation Scale (mCRES) [22]. mCRES is a composite score based on an ordinary grading scale (from 0—normal—to 2) assessing 8 items (thrombocytosis/polycythaemia, organomegaly, adenopathy, endocrinopathy, skin alterations, peripheral oedema, effusions, lung function), for a total maximum score of 16. These scales and scores were also used to define a clinically significant relapse/progression in one of these two scales. To understand the extent of clinical and neurological improvement, we calculated the difference in ONLS and mCRES scores from baseline to the best scores reached after therapy (ΔONLS and ΔmCRES, respectively).

### Definition of clinically relevant sVEGF level change

Duration of sVEGF monitoring was calculated as the time from first institutional visit to last follow-up. sVEGF response after treatment was classified in three different categories: (1) complete response (CR_VEGF_) as normalisation of sVEGF levels (less than 1000 pg/ml) in at least two consecutive occasions; (2) partial response (PR_VEGF_) if a ≥ 50% reduction compared to pre-treatment levels, without normalisation, was observed; and (3) no response (NR_VEGF_) was assigned when neither these two conditions were met [1]. A new significant rise in sVEGF levels was defined as an increase of sVEGF levels (> 1000 pg/ml) in case of previous normalisation or as an increase of ≥ 50% from the lowest level in case of partial or no response to previous therapy on two consecutive occasions [1].

### Statistical analysis

Data are expressed in numbers (%) or median (range). Descriptive parameters were compared by the Chi-squared or non-parametric tests, where appropriate. Events were recorded in case of progression/relapse or death. For time-to-event outcomes, the Kaplan–Meier method was used to estimate relapse-free survival for each group, and the difference between groups was compared by the log-rank test. The HR and 95% Cis were estimated by Cox proportional hazards model. Significant *p*-values, set at < 0.05, and their 95% confidence intervals (CI) were reported. SPSS (SPSS Inc., USA) version 26 was used for all analyses.

## Results

### Study population

Thirty patients regularly monitored for sVEGF were included in the study (Table 1). The median age of onset was 54 years (range 34–71), and there was a male predominance (60%). At inclusion, patients had a moderate to severe neuropathic disability (median ONLS 5, range 1–9) and multisystemic involvement (median mCRES 8, range 1–13). sVEGF level was abnormal at diagnosis in 29 out of 30 (97%) patients (median 3313 pg/ml; range 1042–13,488). sVEGF levels significantly correlated with ONLS scores (*r* = 0.59, *p* < 0.001), but not with mCRES score (*r* = 0.20; *p* = 0.37). Median follow-up was 65 months (9–209), with a median regular sVEGF monitoring of 50 months (6–178).

**Table 1:** Demographic and clinical characteristics

At diagnosis	Total (*n* = 30)
Sex (Male), n (%)	18 (60%)
Age of onset (y), mean (range)	54 (34–71)
ONLS, mean (range)	5 (1–9)
mCRES, mean (range)	8 (1–13)
Elevated sVEGF levels^a^, *n* (%)	29 (97%)
sVEGF levels, mean (range)	3313 (1042–13,488)
At first visit at our clinic	
Treatment-naïve, *n* (%)	22 (73%)
After relapse, *n* (%)	8 (27%)
At last follow-up	
Time on follow-up (m), mean (range)	65 (9–209)
sVEGF monitoring (m), mean (range)	50 (6–178)

^a^ > 1000 pg/mL. *m*, months; *n*, numbers; *sVEGF*, serum VEGF; *y*, years

At the time of presentation to our institution, 22 patients (73%) were treatment-naïve while 8 (27%) had been referred from other centres after a relapse. Autologous stem cell transplant (ASCT) was the most common therapy in treatment-naïve patients (10/22, 45%), followed by radiotherapy (7/22, 32%), lenalidomide + dexamethasone (4/22, 18%), and melphalan + dexamethasone (1/22, 5%). In relapsed patients, lenalidomide was the most common therapy (6/8, 75%), followed by radiotherapy (2/8, 25%). A total of 42 treatment lines were performed in the 30 patients during follow-up, with 29 (69%) resulting in clinical improvement and prolonged remission.

### Utility of regular sVEGF monitoring

#### Treatment followed by remission

**Fig. 1:**
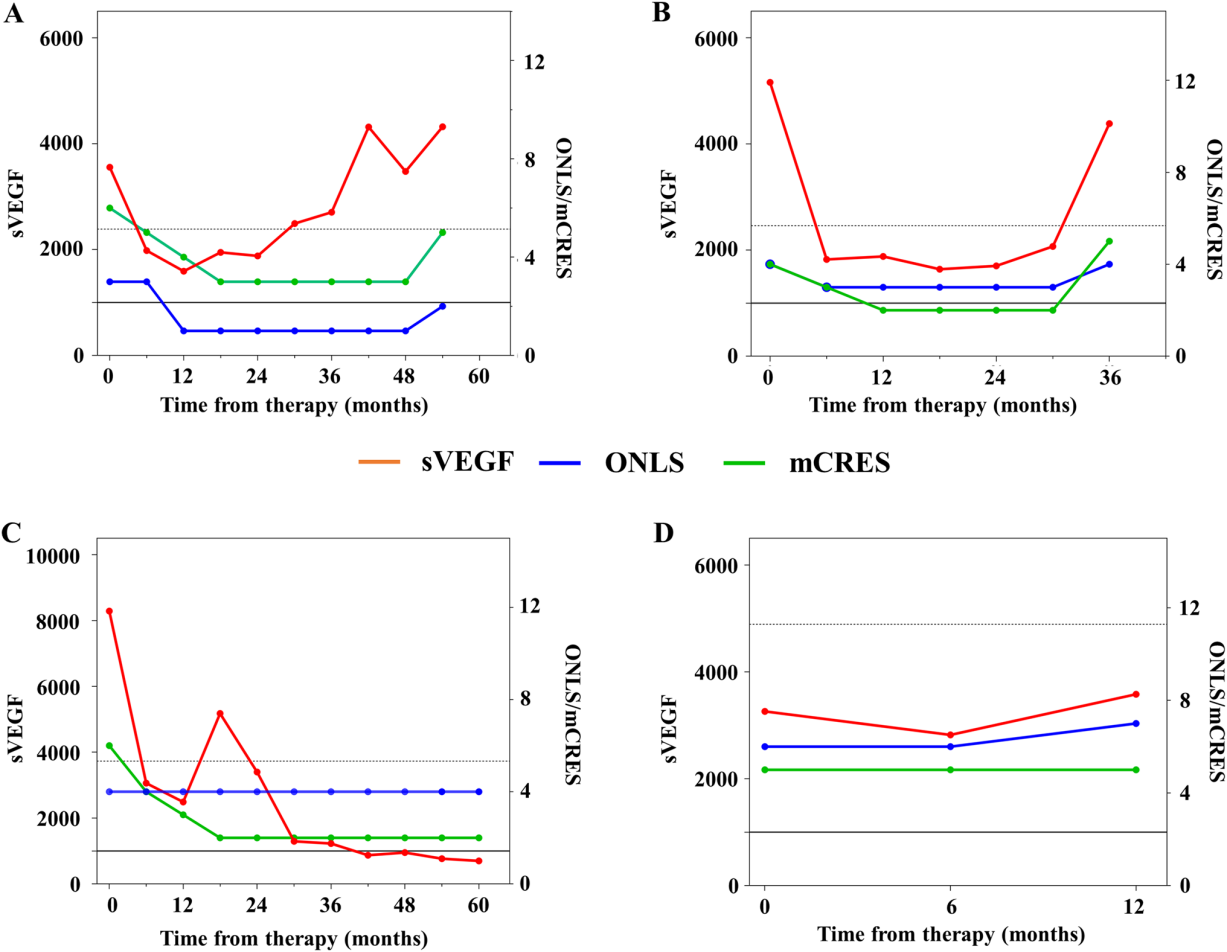
Examples of sVEGF changes (red) in relation to clinical (green) and neurological (blue) scores during follow-up. **A** sVEGF rise anticipating clinical worsening. **B** Pathologic sVEGF rise occurring only at time of clinical relapse. **C** A case developing disease worsening in the absence of significant sVEGF rise. **D** Criteria for sVEGF progression were met in a patient with negative follow-up while on treatment with lenalidomide

Twenty-nine treatments had a prolonged clinical improvement lasting a median of 59 months (95% CI 37–80). sVEGF level was abnormal 6 months after therapy in 18 patients (62%) (median 2100 pg/ml; range 1168–8948), slightly more frequent in cases on active chemotherapy-based regimen (*n* = 9/18, including 7/18 cases treated with lenalidomide) than in cases treated with either ASCT (*n* = 6/18) or RT (*n* = 3/18), and in 13 patients (45%) at 1 year (median 888 pg/ml; range 177–8798), with similar proportion between patients treated with ASCT (*n* = 6/13) and lenalidomide (*n* = 5/13), followed by RT (*n* = 2/13). Twelve of these treatments were followed by a relapse with sVEGF raise anticipating relapse in 7 (58%) by a median of 32 months (range 12–36), being persistently elevated until relapse in all but one patient who had a transient partial reduction in one assessment (Fig. 1A). In three patients, sVEGF was elevated the day when clinical relapse was observed (Fig. 1B), while in two patients, sVEGF levels had not changed despite clinical worsening (Fig. 1C). In the 17 treatment lines without clinical relapse (median follow-up 45 months; range 16–173), sVEGF did not consistently changed during follow-up with only 2 exceptions, where a transient increase was only observed in one measurement (Fig. 1D).

#### Treatment not followed by remission

Thirteen treatment lines were not followed by clinical response with a subsequent progression that in three patients lead to death for massive pleural effusion, ascites, or sepsis. sVEGF level remained abnormal at 6 months after therapy in 9/13 (69%) treatments (median 2100 pg/ml; range 1252–4172), mostly in cases treated with RT (*n* = 6/9), followed by lenalidomide (*n* = 3/9). In 4 patients (31%), there was a significant raise in sVEGF that was associated with further clinical worsening. In two of them (15%), sVEGF predicted further clinical worsening 6 and 12 months before, with constant raising levels after an initial stability after treatment, while in two patients, sVEGF levels were only increased when clinical worsening was clinically confirmed, previously being persistently high but stable. Among the 9 patients without a raise in sVEGF raise, sVEGF levels remained elevated after treatment without further increase at the time of clinical worsening in 5, while in 4 cases, sVEGF levels normalised after treatment despite no clinical response and did not increase at the time of clinical worsening.

#### Remission versus refractory

Therapeutic lines followed by remission differed from those followed by refractory disease in the frequency of PR_VEGF_ at 6 months after therapy (12/29 vs 1/13, p = 0.03) but not in CR_VEGF_ at 6 months after therapy (10/29 vs 4/13, *p* = 0.81).

#### Relapse prediction

**Fig. 2:**
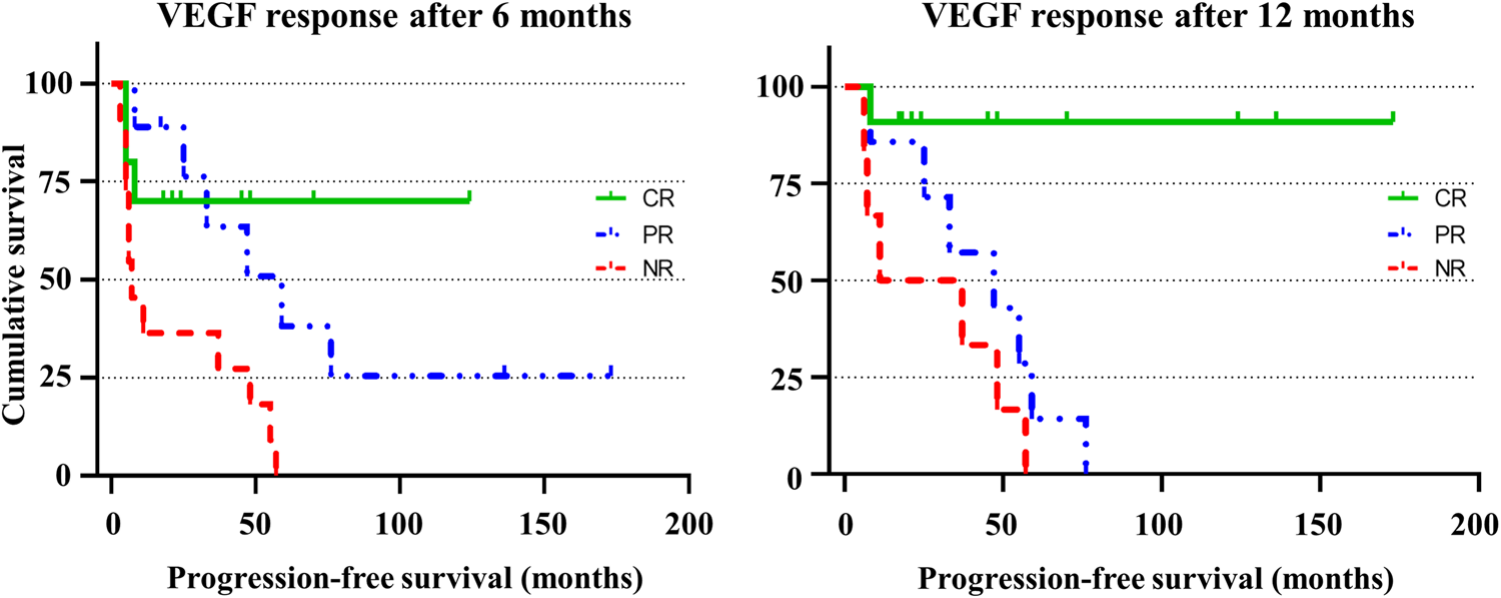
Kaplan–Meier survival curves of patients with POEMS syndrome treated at study entry divided according to sVEGF response after 6 months (**A**) and after 12 months (**B**) after therapy. A complete sVEGF response after 6 months was significantly associated with better relapse-free survival compared to no sVEGF response (CR_VEGF_ vs NR_VEGF_, *p* = 0.015) but there was no difference with partial sVEGF response (CR_VEGF_ vs PR_VEGF_, *p* = 0.61). Instead, a CR at 12 months showed a longer relapse-free survival compared to both no or partial sVEGF (CR_VEGF_ vs NR_VEGF_, *p* = 0.001; CR_VEGF_ vs PR_VEGF_, *p* = 0.005)

Overall, the sensitivity of 6-month sVEGF monitoring in predicting clinical relapse during remission was 58%, while its sensitivity in predicting further clinical worsening during refractory disease was 15% (*p* = 0.025). When we also considered the cases where sVEGF levels were found increased only at the time of the visit revealing relapse or clinical deterioration, sensitivity of the 6-month sVEGF level monitoring was 83% and 31%, respectively (*p* = 0.008). Specificity of the 6-month monitoring of sVEGF levels in predicting clinical relapse during remission was 100%. Remission occurred after 73% of the treatment lines followed by CR_VEGF_ at 6 months, 92% of those followed by PR_VEGF_, and 43% of those followed by NR_VEGF_. The Kaplan–Meier estimates of relapse-free survival at 3 years were 73% after treatment followed by CR_VEGF_ at 6 months after therapy and 66% of those followed by PR_VEGF_ (*p* = 0.61). Remission occurred after 94% of treatment followed by CR_VEGF_ at 12 months, 90% of those followed by PR_VEGF_ (versus CR_VEGF_, *p* = 0.7), and 50% of those followed by NR_VEGF_ (versus CR_VEGF_, *p* = 0.01). On Kaplan–Meier analysis, relapse-free survival at 3 years was 94% after treatment followed by CR_VEGF_ at 12 months, and 57% after the treatment lines followed by PR_VEGF_ at 12 months post therapy (*p* < 0.001) (Fig. 2).

A Kaplan–Meier analysis comparing the time of sVEGF remission before a new raise appear between patients treated with ASCT vs other therapies showed that sVEGF normalisation lasted shorter in the ASCT (median 24 months) group compared to other therapies (median 58 months), albeit the difference was not significant (*p* = 0.114).

In those who achieved remission after therapy (*n* = 29), there was a greater later clinical improvement from baseline in those with CR_VEGF_ at compared to those with either PR_VEGF_ (median ΔmCRES -4 vs -5, *p* = 0.019) or NR_VEGF_ (median ΔmCRES -2 vs -5, *p* = 0.006). Instead, no differences were found in the extent of improvement for neurological scores (median ΔONLS CR_VEGF_ -2 vs PR_VEGF_ -2 vs NR_VEGF_ -1.5, *p* = 0.8).

There was no significant correlation between sVEGF levels and ONLS scores (*r* = 0.28; *p* = 0.1) and mCRES (*r* = 0.27; *p* = 0.1) 6 months after treatment, while at 12 months after therapy, there was a tendency towards a significant correlation for sVEGF levels with ONLS (*r* = 0.41, *p* = 0.053) and mCRES scores (*r* = 0.36, *p* = 0.087).

## Discussion

The utility of sVEGF in the diagnosis of POEMS syndrome and its distinction from other paraproteinemic and demyelinating neuropathies has been established in large cohorts of patients by different studies [10–12, 25] and major diagnostic criteria for POEMS syndrome include increased VEGF [1, 25]. sVEGF levels usually decrease following successful therapeutic intervention and are predictive of clinical improvement and outcome [13, 14, 20–22]. Symptomatic worsening is usually accompanied by raising VEGF levels [13, 14, 26], even if some authors warned that a VEGF raise may be absent despite overt clinical relapse/progression and vice versa [15, 16], leaving some doubts on the utility of sVEGF testing to monitor potential new disease flares.

Our study supports the role of sVEGF as a marker of disease activity, treatment response, and prognosis in POEMS syndrome. It also demonstrates that 6-month monitoring of sVEGF can be used to predict relapse in patients with POEMS syndrome after therapy and throughout follow-up. Most of our patients with a NR_VEGF_ at 6 months post therapy relapsed during follow-up, whereas this occurred only in a minority of patients with CR_VEGF_. Moreover, patients with CR_VEGF_ at 6 months post treatment showed significantly longer relapse-free survival compared to those with a PR_VEGF_ and NR_VEGF_ at 6 months post therapy. These findings indicate that reduction in sVEGF may be used to predict response to treatment in POEMS syndrome and to identify early patients at high risk of relapse [13, 14, 20–22].

In our patients, the 6-month monitoring of sVEGF levels made a useful contribution to the prediction and diagnosis of relapse throughout follow-up. The predictive value of a rise of sVEGF for subsequent relapse had a sensitivity of 58% and a specificity of 100%. In two patients, a random fluctuation of sVEGF levels without evidence of disease activity was observed, but this occurred only in a single measurement, suggesting that persistently raised levels rather than transient fluctuations are indicative of ‘true changes’. As a consequence, patients in remission experiencing a rise in sVEGF should be followed more closely and possibly with an expanded battery of exams (e.g. FDG-PET) [1], to detect clinical relapse at an early stage. An early therapeutic change in these patients seems also advisable [14] after careful exclusion of alternative causes [11] giving the absence of patients in our cohort who maintain persistently elevated sVEGF levels without relapsing.

In 25% of the patients in remission, elevated sVEGF values were observed only at the time of relapse, but not 6 months before. If we assume sVEGF does not rise suddenly but increase progressively, it is likely that a more frequent sVEGF assessment might provide a greater predictive accuracy.

In patients with refractory disease, the 6-month sVEGF monitoring had little value in the prediction and diagnosis of further clinical worsening, with a sensitivity of 15% and 31%, respectively. In most of these patients, however, sVEGF values remained persistently elevated after therapy until subsequent clinical worsening, suggesting that regular sVEGF monitoring may help to confirm non-response to therapy with the need of a therapeutic change.

Even if the main limitation of our study is its retrospective design and the relatively small sample size of patients investigated, it confirms that the 6-month assessment of sVEGF is useful to monitor treatment response and to predict relapses in POEMS syndrome. The appearance of a significant rise in sVEGF before overt clinical worsening may lead a closer clinical assessment and early therapeutic intervention. Future prospective studies may help to further define the timing of VEGF measurement during follow-up of patients in remission and refractory disease.
